# SHARING THE CARE: PAID AND FAMILY CAREGIVERS WORKING TOGETHER TO SUPPORT OLDER ADULTS AT HOME

**DOI:** 10.1093/geroni/igae098.1691

**Published:** 2024-12-31

**Authors:** Jennifer Reckrey, Regina Shih

**Affiliations:** Chair; Discussant

## Abstract

Collaboration between paid caregivers (e.g., home care workers) and family caregivers (e.g., spouses, adult children) is essential to ensure that functionally impaired older adults can thrive at home. However, there are few evidence-based practices and policies to support collaboration and paid and family caregivers are frequently left to navigate shared care on their own. In this symposium, we describe ongoing efforts to identify, understand, and promote collaboration within the paid and family caregiving team. First, Scales et al. describe the development and evaluation of a caregiving policy agenda that brings together diverse partners to support both paid and family caregivers. Miller et al identify trajectories of paid and family care among older adults in the last years of life and describe increased reliance on family caregivers among those living in rural areas. Fabius et al characterize notable role-sharing between paid and family caregivers, particularly among individuals with dementia or enrolled in Medicaid. Reckrey et al share national data that describes the growing prevalence of family members who are themselves paid to provide care, frequently to older adults with high care needs. Finally, Ornstein et al. outline the conceptualization and development of a large caregiving study that draws from the principles of relational coordination to simultaneously examine paid and family care among a multisite cohort of 4,000 older adults. Taken together, these abstracts underscore how research inclusive of both paid and family caregivers lays the foundation for stronger, more collaborative care for older adults at home. Paid Caregiving Interest Group Sponsored Symposium

